# Supplementary figures and images for: The CLASS (Cerebral visual impairment Learning and Awareness for School Staff) Pilot Study: An evaluation of the awareness of CVI amongst teachers and comparative evaluation of two different educational resources on understanding
Source: PLoS One. 2025 Jun 9;20(6):e0324914. doi: 10.1371/journal.pone.0324914 (PMC12148153; doi:10.1371/journal.pone.0324914)

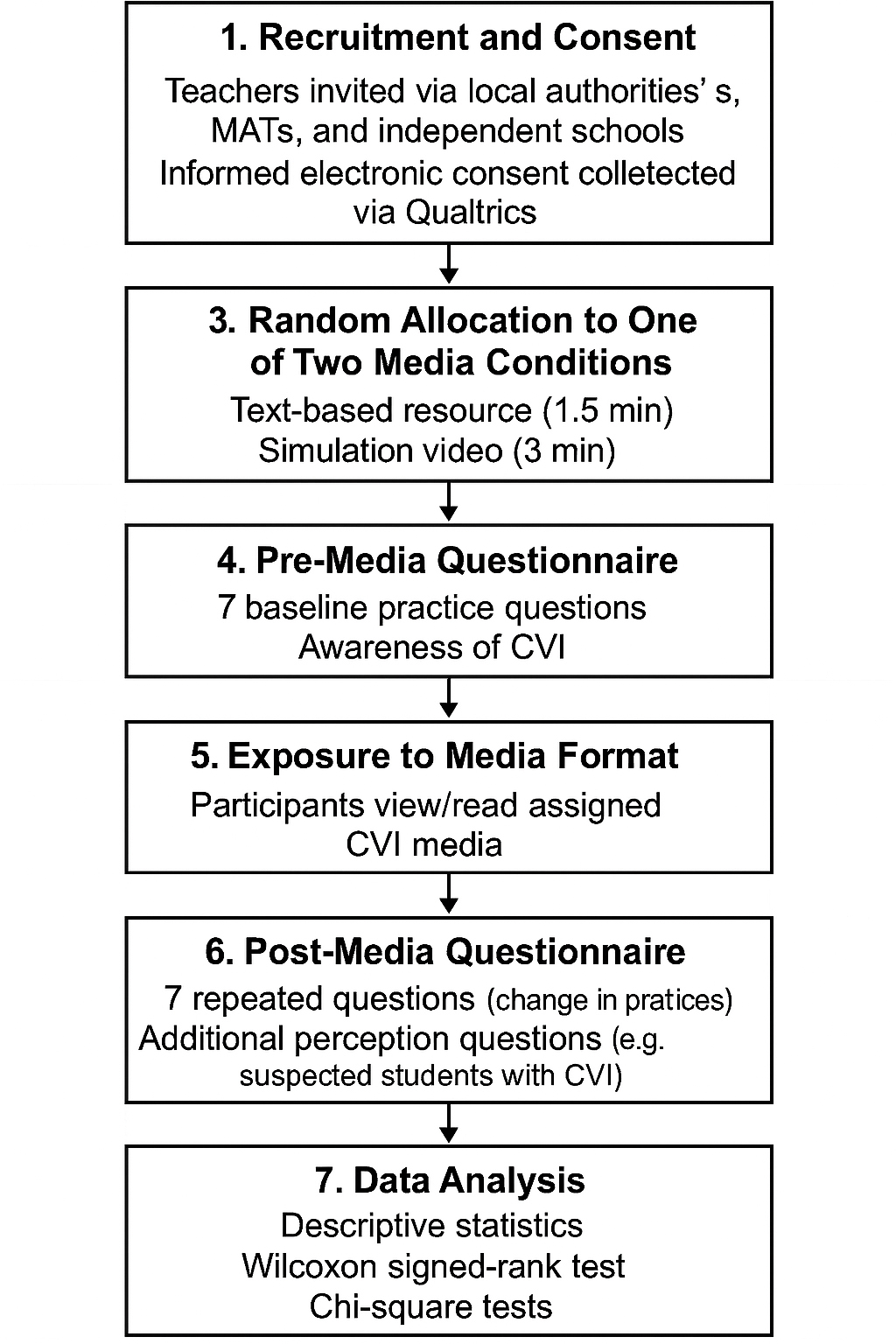

Supplement: S1 Appendix — A visual summary of the research process undertaken in the CLASS pilot study, including participant recruitment, media allocation, pre- and post-media questionnaires, and data analysis procedures. (JPG) [file pone.0324914.s001.jpg]
