# Supplementary material for: The CLASS (Cerebral visual impairment Learning and Awareness for School Staff) Pilot Study: An evaluation of the awareness of CVI amongst teachers and comparative evaluation of two different educational resources on understanding
Source: PLoS One. 2025 Jun 9;20(6):e0324914. doi: 10.1371/journal.pone.0324914 (PMC12148153; doi:10.1371/journal.pone.0324914)
Supplement: S2 Appendix — (PDF) [file pone.0324914.s002.pdf]

Introduction

Hello my name is Aloka Jayasinghe, I am a first year medical student at the University of St Andrews. Thank you for taking the time to complete this survey which should take no longer than 5 minutes to complete.

First, can you please read the Participant information sheet using the link Participant information sheet
If in agreement please click the arrow to proceed.

Block 8

Consent
The University of St Andrews attaches high priority to the ethical conduct of research. We therefore ask you to consider the following points before agreeing to participate in this research. Your agreement confirms that you are willing to participate in this study, however, agreement does not commit you to anything you do not wish to do and you are free to withdraw your participation at any time.

Please click on the following if you agree:

- ☐ I understand the contents of the Participant Information, above
- ☐ I have been given the opportunity to ask questions about the study and have had them answered satisfactorily.
- ☐ I understand that my participation is entirely voluntary and that I can withdraw from the study at any time without giving an explanation and with no disbenefit.
- ☐ I understand who will have access to my data, how it will be stored, in what form it will be shared, and what will happen to it at the end of the study.
- ☐ I understand that if as my information (data) was anonymous at the point of collection, it cannot be withdrawn.

Consent

Having had the opportunity to read both the Participant Information Sheet and Consent Form, do you consent to participate in this study?

- ◯ Yes
- ◯ No

Data collection questions Pre-media Questions

Do you teach in a :

- ◯ Mainstream Primary School
- ◯ Mainstream Secondary School
- ◯ Special School
- ◯ Other (please note below)

If other please note :

Pre-media questions

Before seeing this survey had you ever heard of CVI?

- ◯ No
- ◯ Yes

Before seeing the survey had you ever heard of Cerebral Visual Impairment?

- ◯ No
- ◯ Yes

Before seeing the survey had you ever heard of Cortical Visual Impairment?

- ◯ No
- ◯ Yes

Do you do any of these in your classes?

	Definitely Not	Unlikely	Not sure	Likely	Definitely	Not applicable
Reduce the number of things on the walls in the classroom	◯	◯	◯	◯	◯	◯

	Definitely Not	Unlikely	Not sure	Likely	Definitely	Not applicable
Wear plain clothes without patterns.	○	○	○	○	○	○
Wear similar plain clothes each day	○	○	○	○	○	○
Sit or stand in roughly the same place when teaching	○	○	○	○	○	○
Use children's names when addressing them	○	○	○	○	○	○
Only have one thing at a time on boards and smart screens	○	○	○	○	○	○
Remove unnecessary items from smart screen for example icons and wallpaper	○	○	○	○	○	○

Media - text

Please read the passage below, then click the arrow to the next page where you will be asked a few short questions.
It should take around 1 ½ minutes to read.

Being a teacher of a classroom of children with different needs is hard. In addition to knowing specialist areas, most teachers now need to also understand the support needs of children who face many challenges due to autism, ADHD, difficulties learning, different mental health issues and more. There is also common but widely unknown medical condition that has been associated with all of these difficulties.

Research has shown that there are some children at school who may be struggling due to this condition, but with adjustments to the classroom environment and the way they are taught, it's effects can be reduced. It's called cerebral visual impairment, often shortened to CVI.

CVI is missed because in most cases the child's vision is sharp, and so it is not picked up in standard sight tests. But they still do have a visual impairment. CVI can cause perceptual visual difficulties.

What does that mean? It is probably easier to explain what it may look like, compared to typical vision.

Imagine your classroom from the perspective of the child. What can they see? You, a whiteboard or smart board, pictures on the wall, books, other children and more. All at the same time. A complete view of the immediate world. That's what a child with typical vision sees.

With CVI the child can often only see one thing well at a time, so they can't see you and the board together easily, only one or the other. If there are lots of things on the board, they might only be able to see one of those things at a time. Having to move their sight repeatedly between you, the board and a book on their desk is difficult and tiring and will

slow their learning down. They might lose what they're trying to look at, like you, especially if you move about whilst teaching. Looking and listening at the same time can also be very difficult. If there is extra noise, either from within the room or outside, that can mean they might not see so well. But these added difficulties are all normal for the child with CVI, so they're not likely to complain about it even though they are struggling.

To you, what it may look like is a child who is not paying attention. A child who is easily distracted. A child who can't keep up with the class. The child with CVI will however be working extremely hard to stay focused and keep up. There are many things you can do to help. Reducing the number of things 'to see' on walls will help enormously. A growing body of research has shown that all children study more effectively in classrooms with less on the wall. With CVI, this can help the child see more easily and therefore see more.

If you wear plain clothes, ideally sticking to the same bold colours, you will help the child with CVI not only see more but find it easier to see you and recognise you. Many children with CVI find recognising faces difficult.

Stand or sit in the same place when teaching, rather than move about. If addressing children, always use their names rather than expecting them to know who you mean by looking at them or pointing to them.

When using boards or screens, limit content to one thing at a time. If using a smart screen, ensure everything that is not necessary, for example icons and wallpaper, is not visible.

There may be a child with CVI hidden in your class. These small changes can be transformative to some, opening up a new world of learning opportunities.

Media - video

Please click on the white arrow in the centre of the 3-minute video below to play it. After watching the video, the arrow to proceed to the next page will appear, there you will be asked a few short questions.

0:00 / 2:56

Post-media questions Part 1

Do you think you might teach or have taught children with CVI based on the media you have just seen? If so, approximately how many?

○ None

○ 1

○ 2-5

○ 5-10

○ 10-50

○ 50+

Picking up from earlier questions; having studied the media, how likely are you to now :

	Definitely Not	Unlikely	Not sure	Likely	Definitely	Already Do	Not applicable
Reduce the number of things on the walls in the classroom	○	○	○	○	○	○	○
Wear plain clothes without patterns.	○	○	○	○	○	○	○
Wear similar plain clothes each day	○	○	○	○	○	○	○
Sit or stand in roughly the same place when teaching	○	○	○	○	○	○	○
Use children’s names when addressing them	○	○	○	○	○	○	○
Only have one thing at a time on boards and smart	○	○	○	○	○	○	○

	Definitely Not	Unlikely	Not sure	Likely	Definitely	Already Do	Not applicable
screens							
Remove unnecessary items from smart screen for example icons and wallpaper	○	○	○	○	○	○	○

Comments

Please use the box below if you would like to add any further comments.

Survey Powered By Qualtrics
