# Supplementary material for: The CLASS (Cerebral visual impairment Learning and Awareness for School Staff) Pilot Study: An evaluation of the awareness of CVI amongst teachers and comparative evaluation of two different educational resources on understanding
Source: PLoS One. 2025 Jun 9;20(6):e0324914. doi: 10.1371/journal.pone.0324914 (PMC12148153; doi:10.1371/journal.pone.0324914)
Supplement: Attachment — Submitted filename: Response to Reviewers.docx [file pone.0324914.s005.docx]

**Journal Requirements**

1. **Formatting and File Naming:**
   - The manuscript has been checked against PLOS ONE formatting templates.
   - Files have been named according to guidelines: Manuscript, Revised Manuscript with Track Changes, and Response to Reviewers.
2. **Ethics Statement Placement:**
   - The ethics statement has been moved to the end of the Methods section and includes the approval code: MD16936.
3. **Data Availability Statement:**
   - A revised statement has been included, directing requests to the University of St Andrews School of Medicine Ethics Committee (medresearch@st-andrews.ac.uk) in compliance with PLOS data sharing policy.
4. **Supporting Information:**
   - All references to the Supporting Information have been updated to "S1 Appendix" & S2 Appendix”.
   - A caption has been added at the end of the manuscript under a "Supporting Information" heading.
   - There are now two appendices: **S1**, a newly added flowchart illustrating the research process (as recommended by Reviewer 1), and **S2**, which contains the questionnaire, consent form, participant information sheet, text-based media script, and debrief.
5. **References:**
   - References 1 and 2 have been reformatted to align with Vancouver style.
   - Several online/media citations (References 25–28) have been updated for clarity and consistency.
   - Reference 28 has been updated to the published version of the iLRN 2024 conference paper.
6. **Additional Note Regarding Figures:**
   - All revised figure files (Figures 1–5) have been uploaded to the Preflight Analysis and Conversion Engine (PACE) at https://pacev2.apexcovantage.com/ in accordance with PLOS ONE submission guidelines. Each figure was reviewed and amended to meet the required specifications, then reuploaded via PACE. We confirm that all figures now comply with journal standards and have been submitted through the appropriate platform. Please let us know if any further action is required.

**Editor**

**Editor Comment**

First, ensuring consistent terminology throughout the text is important, especially regarding acronyms such as CVI and phrases like "bitesize learning" and "media format." Defining these acronyms upon first mention in the main text will aid in understanding. Additionally, some paragraphs in the "Results" section are lengthy; consider breaking these down into more digestible segments and adding subheadings where applicable to improve readability.

**Author Response:**
Thank you for these helpful suggestions. We have made the following changes in response:

- **CVI Definition:** The term CVI is defined on first mention in the Introduction.
- **Terminology Consistency:**
  - “bite-size” is used consistently throughout the manuscript.
  - “media format” has been used in place of “media type” for consistency.
  - The term “immersive” has been removed or clarified where appropriate to avoid potential bias.
- **Paragraph Length:** The Results section has been reviewed and revised for readability; several long paragraphs have been broken into shorter, more digestible sections.

**Continuity and Presentation Order:**
To improve consistency and reader clarity, we have standardised the order of school types throughout the manuscript, tables, and figures. References to participant groups now consistently follow the order: **Primary, Secondary, Special, Other**.

**Editor Comment:**
In the discussion section, expanding on how your findings can be integrated into existing teacher professional development programs would be beneficial. Providing concrete recommendations or policy suggestions will strengthen the practical implications of your research.

**Author Response:**
Thank you for this helpful suggestion. We have added a new paragraph to the Discussion section, highlighting how this study could inform the development of professional development resources for teachers. As the first study to systematically measure teacher awareness of CVI, our findings demonstrate an urgent need to raise awareness—particularly in mainstream schools, where CVI remains largely unknown. Without broader awareness, the inclusion of CVI in formal teacher training programmes remains unlikely. While our study was limited to a six-week data collection window due to the constraints of a summer research scholarship, it serves as a strong foundation for further work.

The CLASS materials tested in this study are themselves effective awareness-raising tools and could be embedded into school-based training sessions or local authority CPD frameworks. We have also acknowledged that teacher training in the UK is often school- or authority-led, highlighting the importance of influencing practice through accessible, evidence-based resources. Additionally, we point to the issue of increasingly cluttered classroom environments, which poses particular challenges for children with CVI. Based on our findings, we propose practical, policy-relevant strategies such as encouraging the reduction of unnecessary visual stimuli in classrooms. These points are now addressed in the revised Discussion section.

**Manuscript Addition**

***Beyond questions of scale and format, this study also provides a foundation for influencing how CVI is approached within teacher professional development.*** *As the first study to systematically measure teacher awareness of CVI, it highlights a fundamental barrier to inclusion: without basic recognition of the condition, particularly in mainstream schools, CVI is unlikely to be addressed in training programmes or classroom practice. While our research was time-limited by the six-week window of a summer scholarship scheme, it demonstrates that even short, accessible materials can spark awareness and willingness to change. The CLASS resources trialled here could be embedded within school-based training or local authority CPD frameworks. As professional development in the UK is often shaped at school or regional level, providing teachers with evidence-based, ready-to-use resources is essential. A further policy implication lies in the increasingly cluttered visual environments of classrooms. With mounting displays, labels, and decorations, visual overload is now common, and our findings support clearer guidance to reduce distractions and promote inclusive visual design—benefiting all learners, especially those with CVI.*

**Reviewer 1**

**Reviewer 1 Comment:**
I recommend that the introduction section provides a more detailed overview of teachers' current understanding of Cerebral Visual Impairment (CVI) and emphasizes the important social significance of enhancing teachers' awareness of this condition.

**Author Response:**
Thank you for this comment. We have clarified in the Introduction that, to our knowledge, no prior research has systematically evaluated teacher awareness of CVI—our study is the first to address this gap. The very low levels of awareness identified (e.g. 98% of primary teachers had never heard of CVI) support this assertion and highlight the urgent need for improved education and training. We have also expanded our discussion of the broader social significance of teacher awareness, noting that undiagnosed CVI can contribute to misunderstanding, stigma, and exclusion in classroom environments. Without informed recognition, children with CVI may be misperceived as inattentive, poorly behaved, or less capable, leading to inappropriate responses and missed opportunities for support. These points are now more clearly articulated in the revised Introduction.

**Manuscript Addition**

*Low awareness of CVI among teachers can have important social implications. Without a clear understanding of the condition, children’s behaviours may be misinterpreted, for example as inattentiveness or disengagement, rather than recognised as indicators of unmet access needs. These misunderstandings can unintentionally lead to reduced support, missed learning opportunities, and social exclusion, particularly in mainstream education settings, where teachers are already navigating a wide range of learning needs.*

**Reviewer 1 Comment:**
To ensure that this study is representative, could you further elaborate on the criteria and methods used for selecting the research population? Additionally, please clarify the approach taken to choose the participating schools and how the sample size was calculated.

**Author Response:**
Thank you for this comment. We aimed to recruit a minimum of 382 teachers across the UK, based on a target of 95% confidence with a 5% margin of error from the overall teacher population of approximately 650,000. Our recruitment strategy sought to include primary, secondary, and special school teachers from all four UK nations. However, as described in the manuscript, we encountered substantial challenges navigating varied recruitment policies across local authorities, academies, and independent schools. These issues significantly affected participation and are discussed in detail in the Methods section, as we believe they reflect important structural barriers to conducting educational research in the UK. While our final sample fell below the original target, it included a broad range of teachers from diverse school types and settings. This study forms part of a larger, ongoing programme of work that is now extending recruitment across the UK.

**Reviewer 1 Comment:**
I suggest creating a flowchart to clearly illustrate the research process, which would enhance the clarity of your methodology.

**Author Response:**
Thank you for this excellent suggestion. We have created a flowchart illustrating the key stages of the research process, including participant recruitment, random allocation to media conditions, questionnaire structure, and data analysis. This has been included as **S2 Appendix** in the revised submission and is referenced in the Methods section to support clarity and transparency.

**Reviewer 2**

**Reviewer 2 Comment 1:**
Can the authors introduce a section on analysis in the methods?

**Author Response:**
Thank you very much for this helpful suggestion. We agree that a clear explanation of the analytical approach adds value and improves transparency for the reader. We have now added a paragraph at the end of the **Materials and Methods** section describing the statistical tests used (Wilcoxon signed-rank and chi-square), the rationale for using nonparametric methods for Likert-scale data, and the general analytical procedure. We greatly appreciate this comment and hope the addition strengthens the clarity of our methodology.

**Manuscript Addition:**
The survey ran for six weeks, and data were exported for analysis. Descriptive statistics were used to summarise teacher awareness and reported practices across school types. Because the survey used ordinal Likert-scale responses, nonparametric tests were selected to assess changes in reported practices. Pre- and post-media responses were analysed using the Wilcoxon signed-rank test, applied independently to each media format. A chi-square test of independence was used to compare awareness levels and estimated prevalence of CVI across teacher groups. The chi-square analysis was performed using standard statistical tools, and the Wilcoxon analysis was supported by a custom script. Statistical significance was set at p < .05.

**Reviewer 2 Comment 2:**
Even though the authors provide details of ethical processes they had to undergo, they do not provide any ethics approval number.

**Author Response:**
Thank you for highlighting this, and we also thank the editor for noting it in their summary. We apologise for the confusion — the ethics approval number was included in the original manuscript, but the full ethics statement had been placed at the end of the manuscript rather than within the Materials and Methods section, as required by PLOS ONE. We have now repositioned the complete statement in the correct location, and it includes the approval code (**MD16936**) as specified. We appreciate your careful reading and hope this resolves the issue.

**Reviewer 2 Comment 3:**
In line 185 to 189, the authors try to present the characteristics of participants. This would have been better presented in a table format, which would have given room to provide more details about them, e.g. years of teaching, gender etc.

**Author Response:**
Thank you for this helpful suggestion. We agree that a table improves clarity, particularly at the start of the Results section. While we did not collect data on gender, years of teaching experience, or region, we have reformatted the participant characteristics into **Table 1**, which now summarises school type, pupil age range, and educational context. We believe this improves readability and appreciate your recommendation.

**Reviewer 2 Comment 4:**
Line 191 to 200, the authors describe the level of awareness and provide bulleted statistics below it. These statistics would have been better presented as a table, since that will give more detail and will help in appreciation of the chi-square analysis.

**Author Response:**
Thank you for another helpful and thoughtful suggestion. We have replaced the bulleted list with **Table 3**, which presents CVI awareness levels by school type. We agree that the table format improves clarity and allows for better appreciation of the chi-square analysis referenced in the surrounding text. We’re grateful for your attention to presentation.

**Reviewer 2 Comment 5:**
The authors can move the description of the scoring of the questionnaire (line 217 to 227) to Methods.

**Author Response:**
Thank you for this suggestion. We have now moved the description of the Likert-scale scoring and response options to the **Materials and Methods** section, as recommended.

**Reviewer 2 Comment 6:**
The removal of item 5 before from subsequent analysis seems a little out of place. All items in the questionnaire contribute to the measure of a specific quantity; to remove an item there must be enough evidence that will suggest its redundancy. The evidence presented of a marginal pre and post score is not a known means of eliminating an item from a questionnaire. The authors may provide a better statistical justification for its removal.

**Author Response:**
Thank you for raising this important point. We agree that removing an item from analysis requires clear justification, and we have now strengthened our explanation accordingly in the Results section.

The primary aim of the questionnaire was to assess change in teachers’ reported willingness to implement CVI-supportive practices, following exposure to media resources. Item 5 (“Using the child’s name when addressing them”) was excluded from comparative analysis due to a clear **ceiling effect**: nearly all participants reported already doing this before viewing either media format. As a result, there was no meaningful opportunity to observe change or media impact. This was not evident at the point of study design, but became apparent upon initial analysis.

The item was not removed because it was redundant in general, but because it did not serve the **analytic purpose** of evaluating change, which was central to our use of the Wilcoxon test. We have now included a paragraph in the Results section that outlines this rationale more clearly. We also note that the finding itself is useful, suggesting that this particular inclusive practice is already well embedded. We appreciate your thoughtful feedback and hope this clarification addresses your concern.

**Manuscript Addition**

*One item on the questionnaire, ‘Using the child’s name when addressing them,’ was excluded from comparative analysis. This decision was made post hoc due to a ceiling effect: nearly all participants reported already doing this prior to exposure to the media, leaving little to no room for measurable change. As the primary aim of the questionnaire was to assess change in reported practice, this item could not meaningfully contribute to the analysis of media effectiveness. While the item was not identified as unsuitable during study design, its exclusion was necessary to maintain analytical validity. The finding itself is nonetheless valuable, suggesting that this particular inclusive practice is already well established among teachers.*

**Reviewer 2 Comment 7:**
The authors often discuss the findings in the Results section. It will be helpful to the reader if this is avoided and all such statements moved to the Discussion section. An example is the discussion in line 305 to 308.

**Author Response:**
Thank you for this helpful observation. We have now reviewed the entire Results section carefully and addressed your point in the following ways:

- The interpretative paragraph previously on lines 305–308 (relating to variation in class sizes and teacher roles) has been **removed from the Results** and appropriately **relocated to the Discussion**. It now follows the paragraph discussing variation in change across teacher groups and contextualises teacher estimates of CVI.
- In the Wilcoxon analysis paragraph, we have **revised interpretive language** (“confirmed effectiveness”) to a more neutral phrasing: “These findings indicate a significant increase...”.
- A sentence in the Results previously concluding with “highlighting a critical gap in awareness among teachers” has been revised to “indicating low baseline awareness of CVI across teaching groups.” The interpretive framing of a “critical gap” now appears more appropriately in the Discussion.
- We have also removed the following interpretive sentence from the Results: “Overall, the findings suggest that increased awareness of CVI may prompt teachers to consider the possibility of CVI in their students...”. A revised version of this point has been integrated into the Discussion where it better supports our reflections on implications for earlier identification.

We appreciate your feedback on this point, which has helped to improve the structure and clarity of our Results and Discussion sections.

**Reviewer 2 Comment 8:**
The authors have indicated the bias that can be introduced by comparing the number of potential CVI cases among the various categories of teachers. Presenting the results as percentages or ratios may help to reduce the impact of the different class sizes.

**Author Response:**
Thank you for this thoughtful suggestion. We agree that comparing absolute numbers across teacher groups can introduce bias due to differences in class size. This issue is noted in the Results and further addressed in the Discussion, where we highlight the considerable variation in teaching contexts, for example, secondary school teachers typically work with significantly more students than those in primary or special settings.

While we had originally aimed for a larger sample that would allow more granular statistical comparison, we encountered substantial **recruitment challenges** due to **policy complexity across regions** and the **limited six-week recruitment window** tied to a student summer scholarship. Given these constraints, we were cautious not to over-interpret subgroup differences.

Interestingly, despite the expected disparity in pupil exposure, the variation between primary and secondary teacher estimates was not statistically remarkable in this sample. As such, we felt it was most appropriate to report the findings transparently without attempting ratio-based adjustments that could suggest a level of precision we could not support.

A clarifying sentence has now been added to the Discussion to explain this decision and note that further stratified analysis will be considered in future phases of the study, which will involve a larger and more representative sample.

**Manuscript Addition**

*Given the modest sample size and scope of the pilot study, we chose not to present these findings as ratios or percentages, to avoid over-interpreting trends across groups. This approach will be re-evaluated in the next phase of research with a larger dataset.*

**Reviewer 2 Comment 8:**
The authors state as one of their objectives “compare the effectiveness of text-based versus immersive virtual reality simulation video resources in increasing CVI awareness.” However, there were no descriptive results that demonstrated this, except for the chi-square results presented. I will suggest the authors present results that segregate responses based on media type.

**Author Response:**
Thank you for highlighting this important point. We agree that segregating responses by media format enhances clarity and supports our stated objective of comparing resource effectiveness. To address this, we have now included a new figure (Figure 5) which presents the average Likert scores for each of the seven questions, broken down by media type and time point (pre- and post-).

To maximise accessibility and interpretability, we used consistent colour schemes for each media format (blue for text-based, purple for video) and distinguished pre- and post-responses using line styles (dotted vs. solid). This figure allows for a clear visual comparison of the magnitude and pattern of change associated with each media type.

As described in the Results section, both formats showed statistically significant gains. The new descriptive figure provides further insight into the direction and consistency of those changes across individual questions. We are grateful for your suggestion and believe this addition strengthens the manuscript.

**Reviewer 2 Comment 9**
Figure 4 and 5 have no x-axis and y-axis labelling.

**Response:**
Thank you for highlighting this oversight. We’ve now added clear x-axis and y-axis labels to both Figures 4 and 5. We appreciate this helpful comment, which has improved the clarity of the figures.

**Reviewer 2 Comment 10**
The information provided in table 2 is the same as in figure 2. The authors may remove one of them.

**Response:**
Thank you for this helpful suggestion. To reduce redundancy while preserving clarity, we have removed Figure 2 and retained Table 2, which presents the same information along with the calculated pre/post differences for each question.
